# Critical Review on Sustainability in Denim: A Step toward Sustainable Production and Consumption of Denim

**DOI:** 10.1021/acsomega.2c06374

**Published:** 2023-01-13

**Authors:** Aravin Prince Periyasamy, Saravanan Periyasami

**Affiliations:** † Department of Bioproducts and Biosystems, School of Chemical Engineering, Aalto University, Espoo02150, Finland; ‡ Thuan Phuong Company, Limited (Garments-Embroideries), Ho Chi Minh City, Vietnam

## Abstract

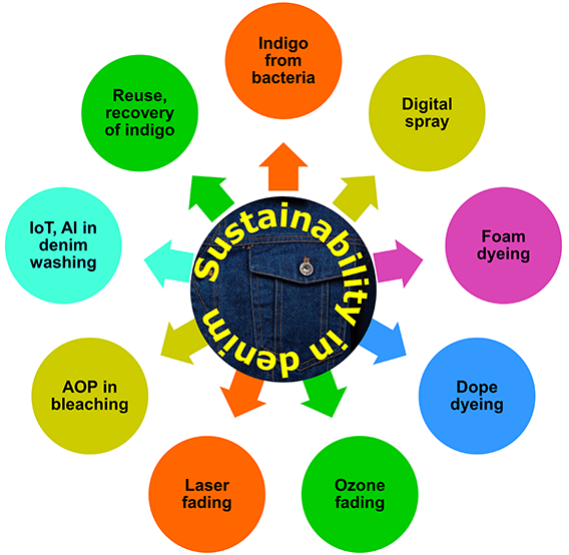

The exponential development in knowledge on the health and environmental concerns linked to conventional denim processing is directly responsible for the continuous increase in demand for the exploitation of sustainable denim. Research is essential to explore alternative methods to reduce the environmental impact caused by these industries. This review examines the many sustainable ways to produce denim, keeping in mind the problems that the denim industry is now facing in finding alternatives to conventional manufacturing practices. The most current advancements in environmentally friendly dyeing techniques for denim have been extensively discussed. These processes include the production of indigo from bacteria as well as different dyeing processes, such as digital spray, microbially assisted dyeing, and foam dyeing denim with indigo. In addition, this review covers the many environmentally friendly finishing methods for denim garments, such as ozone fading, e-flow, enzyme-based bleaching, water, laser fading, and so on. Finally, it is described how the chemical and mechanical processes used to finish denim might affect the amount of microplastics and microfibers released from the denim garment during domestic washing. As a result, the content presented in this review aims to address the importance of sustainable denim processing, that is, something that can be rethought, reevaluated, renewed, and restructured within the scope of conventional denim processes, while taking eco-responsible solutions for increased environmental sustainability into account.

## 1 Introduction

In comparison to other planets in the solar system, Earth stands unique because it has characteristics that are favorable for life and withstands various human activities. The presence of humans on Earth and their contributions, such as the Agricultural and Industrial Revolutions, followed by the complex world of synthetic and man-made materials have a negative impact on biodiversity and the ecosystem. The birth rate is 2–3 times higher than the mortality rate, and the development of medications extends life expectancy from the current population level of 7.6 billion to roughly 9.8 billion by 2050.^1^ The majority of environmental destruction is caused by population growth, which also has an impact on land use and demands for resources like food, water, minerals, and fossil fuels. Global warming and climate change are a result of the exponential growth of deforestation.

The Industrial Revolution has given rise to several industries, including the textile industry, with the denim industry being one of them. There are several chemical treatments used in the denim industry to remove impurities and coloring and to produce appropriate finishing. The coloration process involved utilizing a huge quantity of water and energy, discharging the effluent into the environment, and emitting greenhouse gases (GHG).^2−4^ Approximately 600 000 tons of dyes are produced globally and used in the textile industry including the denim sector; in that sector, azo dyes make up almost one-half of those dyes.^5^

The achievement of the UN sustainable development goals (SDG) by 2030 is significantly influenced by the denim industry. As was already discussed, SDG6 is impacted by the denim industry’s large-scale discharge of contaminated water that contains toxic materials, dyes, and other additives. The manufacture of denim produces higher GHG emissions and is a crucial industry for the climate change described in SDG13. The issue of marine pollution as well as the emission of microfibers and microplastics into the marine environment due to home washing is addressed by SDG14. Additionally, cotton farming has a significant worldwide impact on soil quality and emphasizes SDG15.

Denim is among the most widely used items of apparel since it suits people of all ages, all seasons, and all occasions, resulting in denim becoming a popular fashion item. Denim is produced by using coarser cotton yarns with twill view. By 2027, the market for denim jeans is expected to be valued at around 87.4 billion US dollars, up from 63.5 billion US dollars in 2020.^6^ The worldwide e-commerce business, which is expanding rapidly, has a positive impact on the demand for textiles including denim. The United States is the largest market for denim globally and has the highest per capita consumption of denim. Apart from that, the demand for denim is mostly driven by rising income levels, more fashion consciousness, and the move toward informal attire in the workplace.^7^

For several denim manufacturers in the most recent year, sustainability has evolved into their unique selling point. Energy conservation and emission reduction of green environmental protection principles have become the most fundamental criteria of the denim dyeing business as people’s concern for the environment and resources has continuously risen.^8,9^ Demand for sustainable denim has increased as a result of rising consumer health awareness and a growing understanding of the advantages of wearing organic clothing. As a result, the denim market is predicted to be driven by the introduction of sustainable denim as a popular option of clothing during the forecast period. This review is devoted to discussing the different environmentally friendly processing techniques for denim, including electrochemical reduction, enzymatic processing, plasma, ultrasonic and laser-assisted processing, and an advanced oxidizing process, which enhances the precise properties with minimal environmental impact.

## 2 Denim Supply Chain

**Figure 1:**
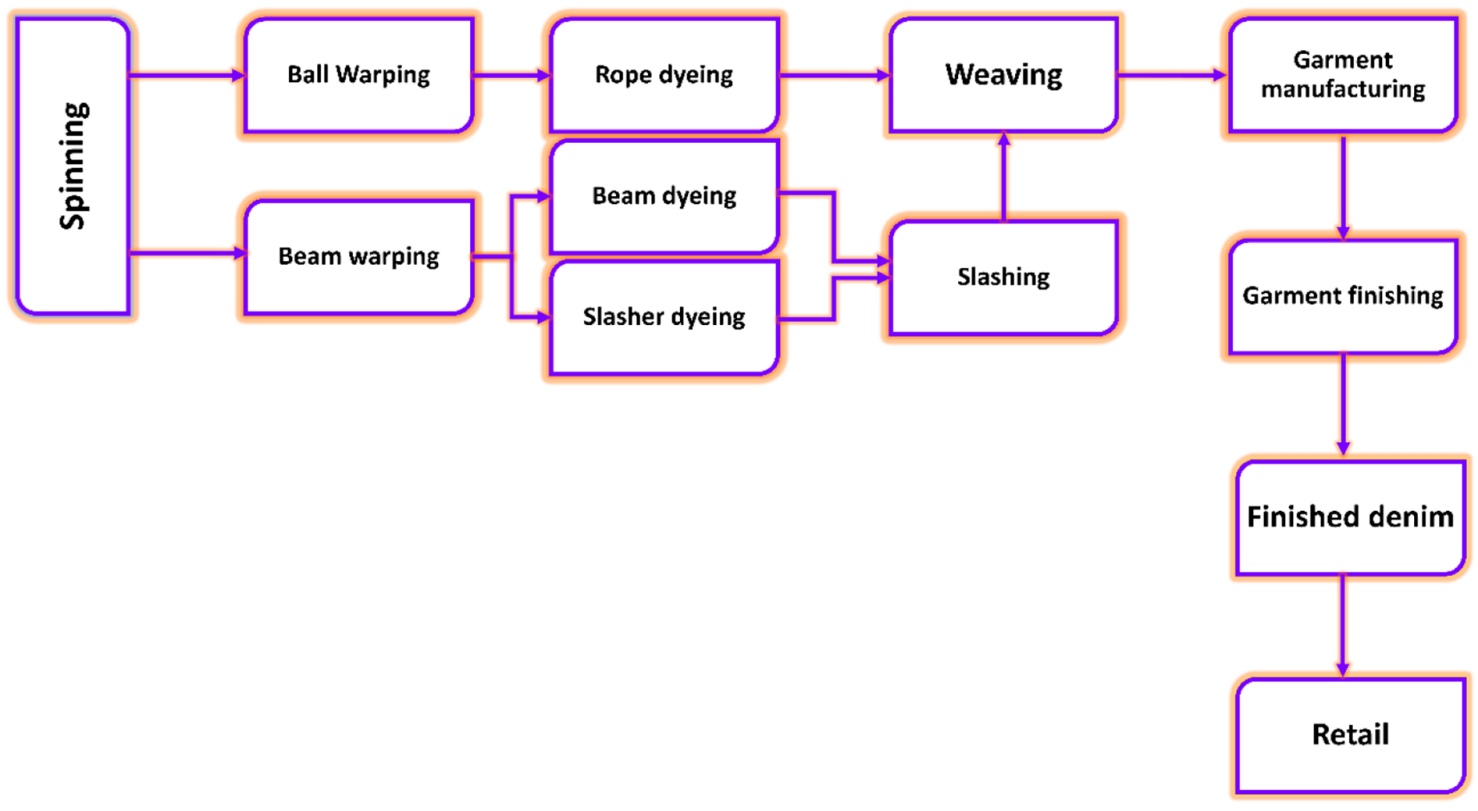
Process sequence for denim manufacturing from fiber to finished garment.

For denim manufacturing, the coarser warps are used in the range of 100–30 Tex than weft. Warp was treated with the indigo dyes, whereas the weft was left undyed. Figure 1 shows the overall manufacturing sequence of denim. Nevertheless, raw material for denim is acquired either naturally or artificially. Henceforth, it is essential to implement the first stage of categorization to cultivate or produce the fibers.^10,11^

The initial step in the spinning process is the blowroom and carding activities to open and clean the cotton fibers from bales. Later, the fibers were formed in slivers in the carding and drawing process to produce yarns from the ring or open-end spinning process.^12,13^ The produced yarns are afterward used for warping to create the fabric. Warp sheets must undergo sizing to increase their strength because of the wear and tear during the weaving process. Since several chemicals are used in the sizing process in amounts ranging from 12% to 15%, resulting in an increasing pollution load.

### 2.1 Coloration of Denim

The production of blue denim often involves the use of indigo as the primary colorant in the dying process. Although it is often considered that indigo has poor quality vat dyes, it is widely used in the denim industry because of the worn-out appearance that it gives to the denim fabric. As shown in Figure S1, the chemistry of indigo dyeing comprises an oxidation–reduction reaction. Overall, the denim industry consumes 50 000 tonnes of synthetic indigo;^14^ however, indigo is inherently water insoluble^15^ and therefore required alkali and a reducing agent to be converted into a soluble form before being applied to denim warps.^15^ As a result, 84 500 tonnes of sodium hydrosulfite and 53 500 tonnes of caustic soda are required each year for denim dyeing.^16,17^ Since 100% fixation of dyes is typically not feasible, unfixed dyes are released into water streams causing turbidity and ecological disruption in addition to being poisonous, carcinogenic, or mutagenic.^18^

### 2.2 Desizing

Desizing is mostly employed to remove adhesive flecks from denim fabrics. Diverse chemicals, such as detergents, Na_2_CO_3_, enzymes, and oxidative substances, can be used to do this. Because all other methods outside enzymes induce fabric deterioration, the majority of companies used enzyme-based desizing.

## 3 Sustainability of Denim; Why It Is Important

**Figure 2:**
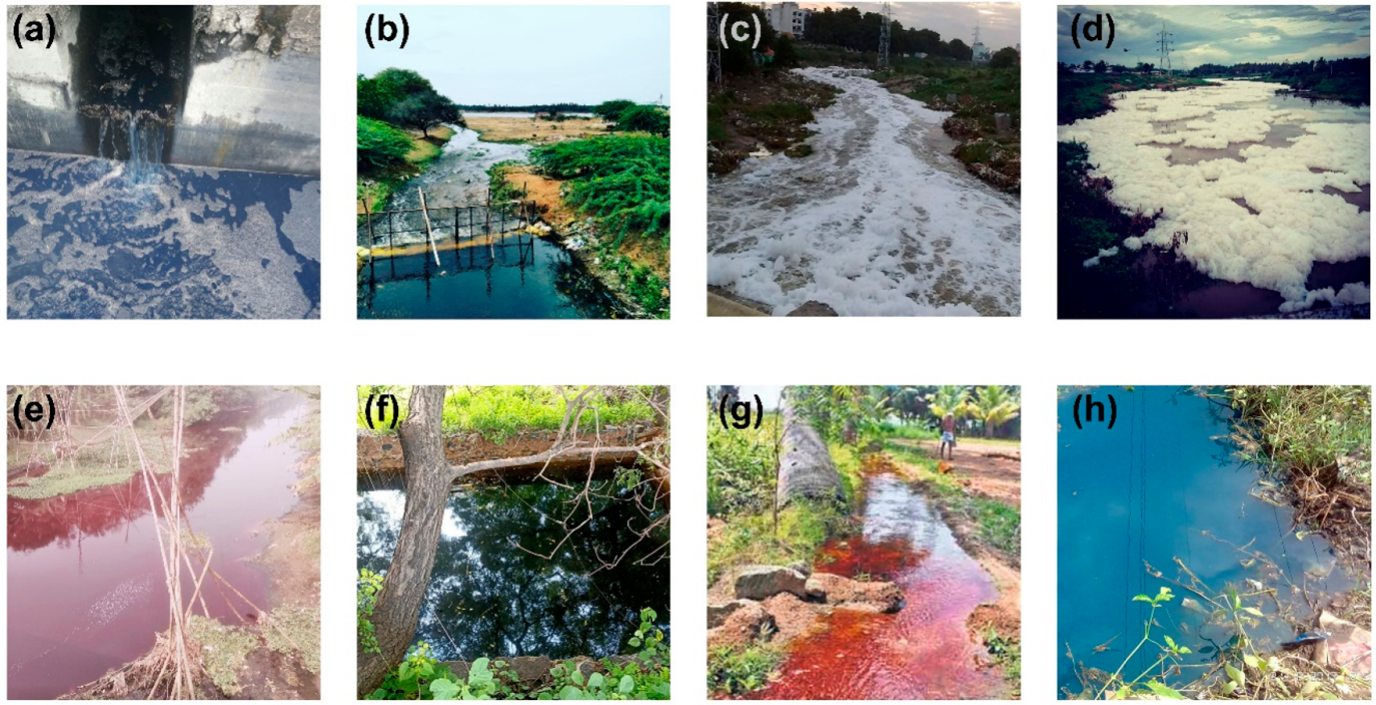
Denim effluent from industry (a) and in the water streams (b) that flow in the Noyal River (c–e) in India, and the influence of these effluents on the agricultural land (f–h) (pictures taken April 2022).

Denim manufacturing results in the release of between 40 and 65 L of effluent per kilogram of denim.^19^ According to Greenpeace International, the production of textiles is responsible for 20% of the world’s water pollution. This problem is especially prevalent in the most populous countries, such as China, India, and Bangladesh.^20^ The wastewaters produced by the denim industry need to be treated before they can be released into the aquatic habitat. In Figure 2, we can see how the denim industries and the wastewater they produce contribute to the contamination of the Noyal River at Tirupur and the agricultural fields in the surrounding area in India. The denim manufacturing process results in the release of colored wastewater (Figure 2a), which then contaminates the streams (Figure 2b) and eventually flows into the river (Figure 2c and 2d). It is clear from the photographs how it is affecting agricultural operations (Figure 2e), particularly the groundwater (Figure 2g and 2h).

**Figure 3:**
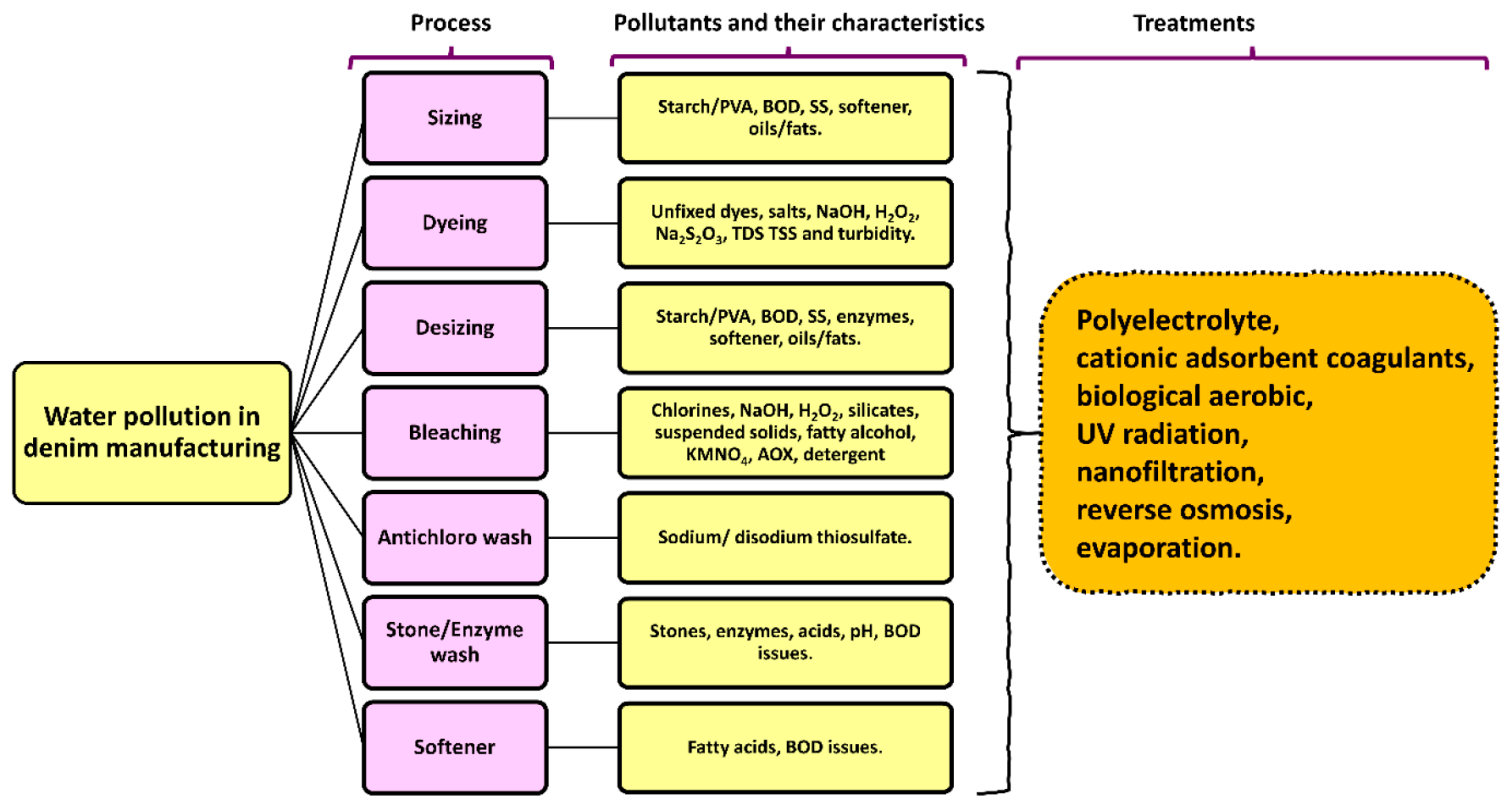
Water pollution during denim processing stages.

Toxic chemicals are used in every stage of the production process, which results in the constant release of a wide variety of hazardous waste into the environment in the form of wastewater. The dyes, auxiliaries, and other chemicals required for the dyeing process are the primary contributors to the pollution. It is known that the dyes contain heavy metals lead (Pb), chromium (Cr), cadmium (Cd), copper (Cu), and nickel (Ni), which are toxic and affect multiple organs in the body, as they affect the kidneys and nervous system and damage the skin and vascular and immune systems; additionally, they cause birth defects and cancer.^21^ During the manufacturing process for denim, the effluents in the wastewater have the characteristics of having a high pH, biological oxygen demand (BOD), chemical oxygen demand (COD), total dissolved solids (TDS), suspended solids (SS), turbidity, chlorides, sulfates, and phenols,^22,23^ which are listed in Figure 3.

These effluents have the most significant impact on the agricultural sector and industry use, which affect day-to-day activities.^24^ The domestic washing process releases microfibers into the environment, and these microfibers contain a variety of toxic elements from the dyeing process, including salts, surfactants, ionic metals, and their complexes, formaldehyde, toxic organic chemicals, biocides, detergents, emulsifiers, and dispersants.^25−27^

### 3.1 Raw Materials and Environmental Concerns

Cotton is possibly the most important natural fiber obtained from cotton plants. According to the Cotton Incorporation study, around 12% of cotton is utilized to make denim garments.^28^ Cotton offers excellent comfort features such as moisture absorption, no static electricity, and a superior feel on the human skin. In terms of landfills and disposal, cotton is a quickly decomposable fabric; yet, it pollutes the land and water owing to excessive fertilizer and pesticide use. Organic cotton may be farmed with less water and fewer fertilizers and pesticides, reducing the environmental effect.

Lyocell is an eco-friendly fiber produced from wood pulp by using the eco-friendly solvent amine oxide. It is manufactured from managed forests and does not require the use of herbicides or pesticides.^29,30^ Lyocell has greater qualities than cotton, such as strong wet strength and luster; thus, it may be employed in denim industries as an alternative to cotton.^31^ Polyester is a cheaper fiber than natural fibers; it has become widely used in a variety of apparel including denim. Furthermore, except for moisture absorption, it has excellent properties similar to natural fibers. More than 70 million barrels of crude oil are consumed each year to create polyester fibers/films and resins.^32^ In terms of environmental considerations, polyester is not biodegradable and remains in the ecosystem for an extended period. Conversely, polyester clothes (i.e., including denim) constitute the primary source of microplastics in the water.^33,34^

## 4 Greener Way To Produce Denim

### 4.1 Synthesis of Bioindigo

It is expected that the global market for synthetic indigo dyes should expand rapidly, reaching a value of 1639 million USD by the year 2028. In the upcoming years, the market is expected to be driven by synthetic indigo because it has a greater tendency than conventional indigos to generate high-contrast fading on jeans.^35^ As a result, it leads to a significant increase in the need for the chemical synthesis of indigo, which is a critical sustainability issue. Aniline was used as a feedstock for indigo synthesis, and additionally, the aniline is derived from benzene, which is considered a toxic chemical. In addition, the synthesis also used formaldehyde, hydrogen cyanide, sodamide, and alkalis, all of which are also toxic.^36,37^

**Figure 4:**
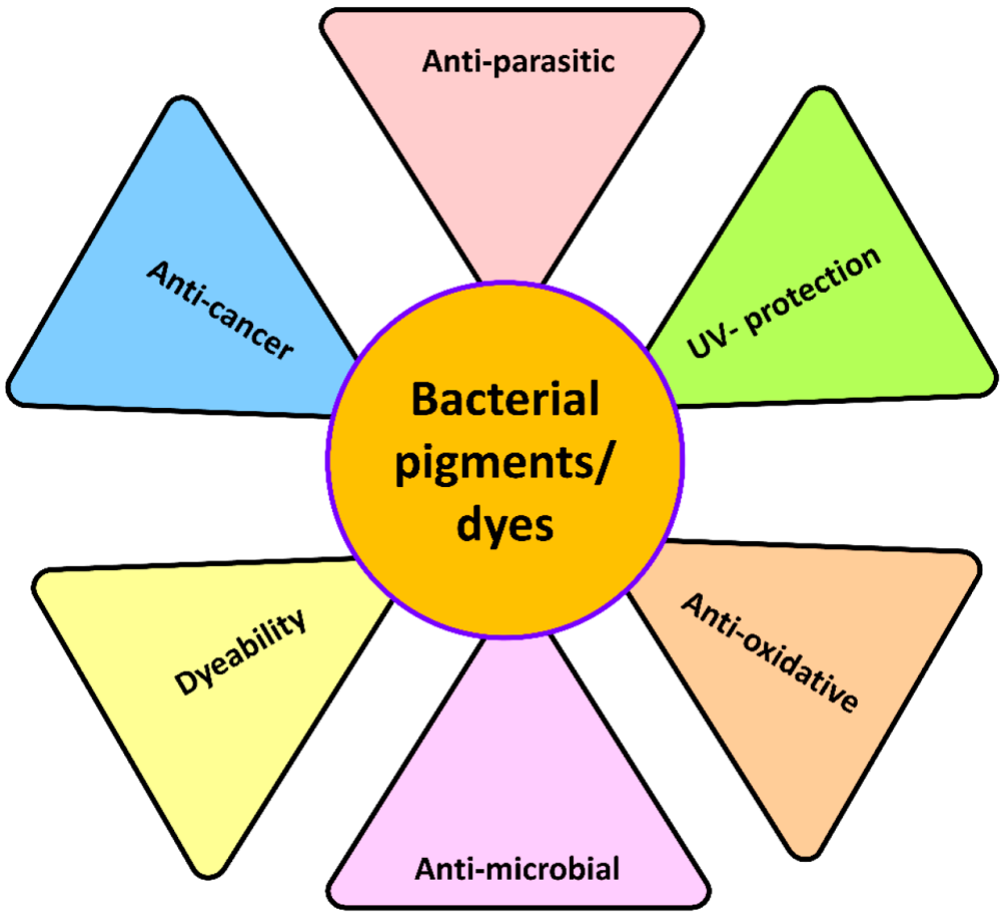
Multifunctional properties of bacterial pigments.

There is a growing need to develop environmentally friendly techniques, and biosynthesis of indigo is one of them.^38−40^ The use of bioindigo could perhaps be better for the environment; because of its biodegradability and low toxicity, bioindigo has gained popularity. When we compare microorganism-produced dyes to plant-based dyes, we see that the range of natural dyes is rather limited.^41^ They demand a lot of growing water and a lot of agricultural surfaces, and they must be harvested, all of which make them more expensive. These compounds can have key additional properties in addition to their color, including potent antimicrobial activity against a variety of pathogens, anticancer activity, antioxidative activity, and UV-repellent properties. Since these pigments provide a wide variety of potential applications in addition to the aesthetic and functional properties that they possess, these pigments might be employed as functional dyes not just for denim but also for other types of textile materials (Figure 4).

**Figure 5:**
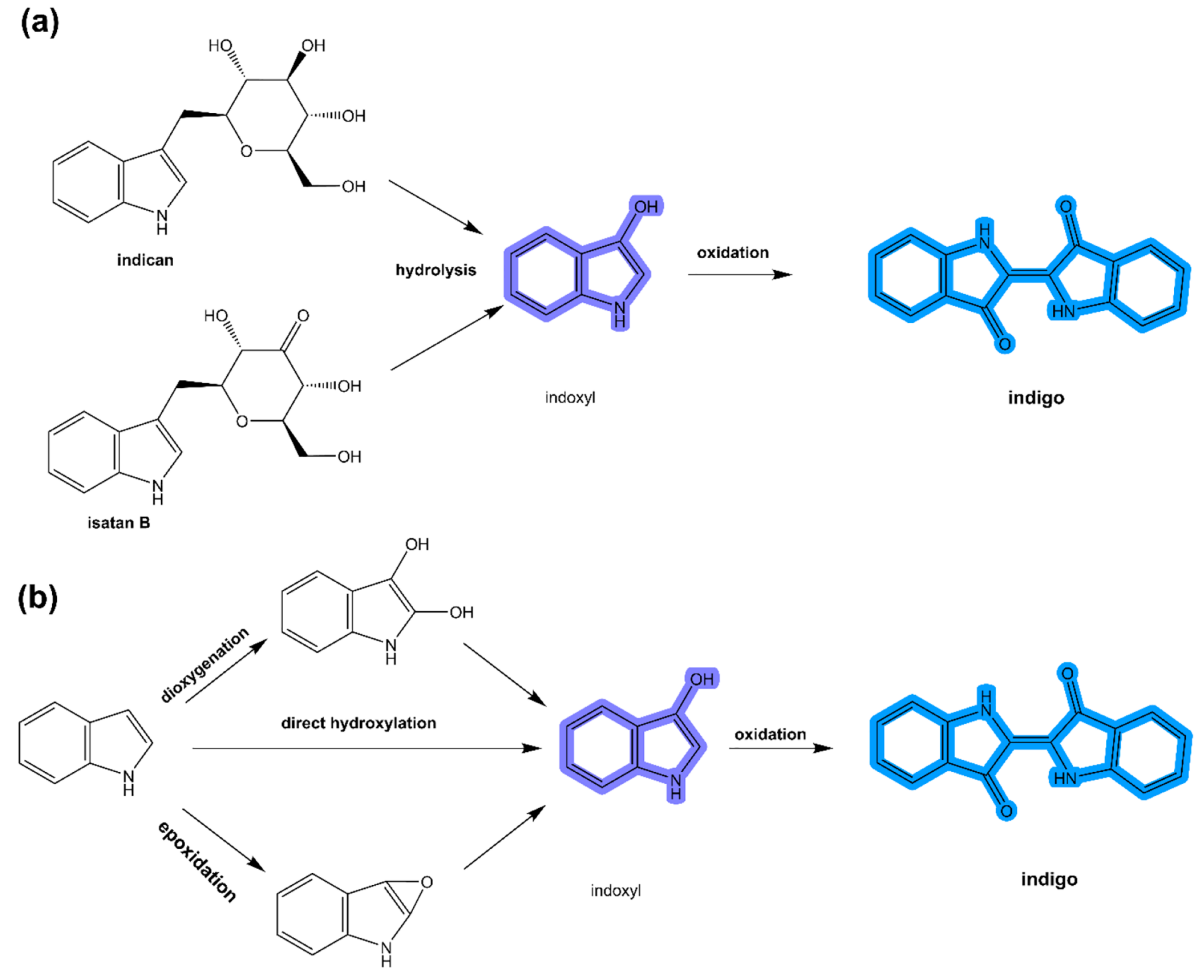
Natural (a) and microbial (b) synthesis of indigo. Adapted with permission from ref (42). Copyright 2019 Springer Nature Limited. Copyright under C.C 4.0 permission.

In recent decades, many indigo-producing enzymes have been found in bacteria. These enzymes all use an oxygenation process to convert indole. Tryptophan or indole might be added to the medium to increase indigo production. This demonstrated that the oxygenase may convert indole into indoxyl, resulting in the production of indigo, and different enzymatic routes such as natural (Figure 5a) and microbial (Figure 5b) synthesis to produce indigo.

**Figure 6:**
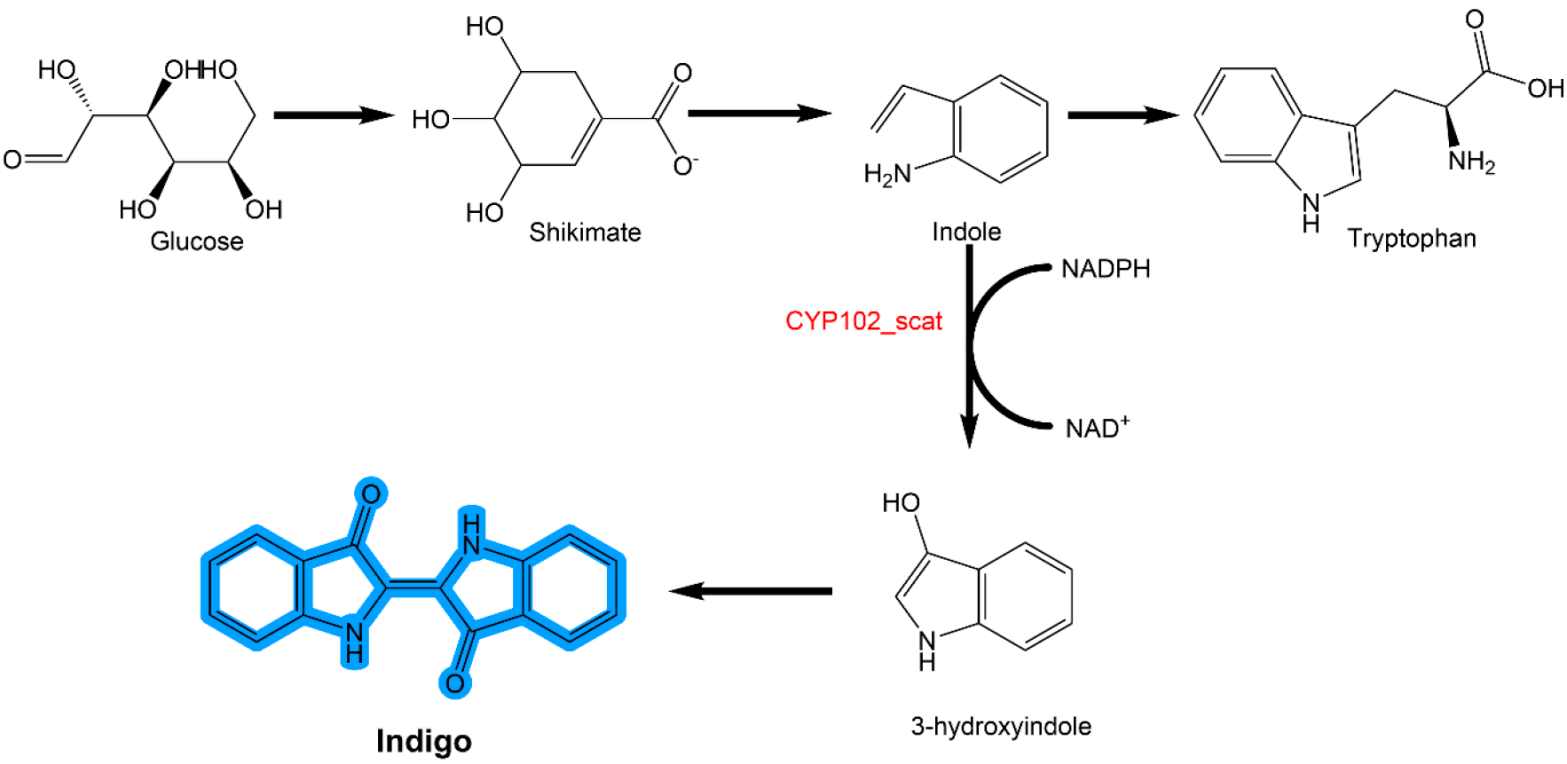
Biosynthesis of indigo from *E. coli* cells. Adapted with permission from ref (43). Copyright 2017 Elsevier.

Tryptophan is a substance that is naturally produced by bacteria and is used in the production of bioindigo (Figure 6). Tryptophan already possesses the ring structure that is essential to the formation of indigo molecules.^38^ This biological technique is fast and simple to conduct when compared to synthetic chemistry-based production methods.^43^ Hsu et al.^14^ proposed the biosynthesis of indican, which was then utilized in the dyeing process for cotton garments. The cotton garments were colored by using concentrated fermentation broth containing 3.2 g/L indican, and it oxidized with atmospheric air. Additionally, they examined the dye with synthetic indigo (i.e., unreduced indigo) at an equimolar ratio; however, there was no evidence of dye penetration or adsorption on the fiber. As a result, indican offers improved dyeing properties and demonstrates total environmental friendliness from synthesis to the cotton textile application.^14^

Recently, there have been some alternative practices for the coloration, such as alkali/salt-free^44−46^ reactive dyeing on cotton. Table 1 provides some information on the alternatives for toxic chemicals. Tia et al.^47^ studied the stability of natural indigo; this research showed that the leuco form of indigo can be stabilized on a nanocellulose matrix carrier without the need for chemical agents, making the dye more ecologically safe to use on cellulosic materials.

**Table 1:** Environmentally Friendly Alternative Chemicals for the Denim Dyeing Process

dyes	traditional	alternatives	refs
sulfur dyes	Na_2_SO_3_	C_6_H_12_O_6_, HSCH_2_CH_2_OH	(48,49)
vat dyes	Na_2_S_2_O_4_, NaOH	electrochemical method	(50−54)
vat and sulfur dyes	K_2_Cr_2_O_7_	H_2_O_2_, [Na^+^]_2_·[B_2_O_4_(OH)_4_]^2–^	(49,54,55)
sulfur, vat dyes		solubilized dyes	
hydrotropic agents	CH_4_N_2_O	NaN(CN)_2_	(56)
neutralizing agents	CH_3_COOH	HCOOH	(56)

### 4.2 Sustainable Dyeing of Denim

**Scheme 1:**
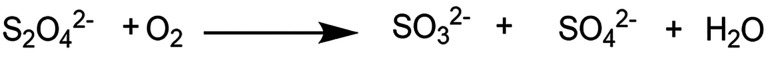
Byproduct Formation of Na_2_S_2_O_4_ during Indigo Dyeing

For the indigo reduction process, many chemicals and approaches have been used. Because of its effective reducing power, ability to reduce indigo at room temperature, shorter duration, availability, ease of handling, and low cost, sodium dithionite (i.e., hydrosulfite: Na_2_S_2_O_4_) is the most often used reducing agent in the industry. Therefore, a larger quantity of sodium hydrosulfite (Na_2_S_2_O_4_) was utilized for the reduction of synthetic indigo since it influences the aerobic processes and has the potential to anaerobically create toxic hydrogen sulfide (SO_3_^2–^) from the sulfate (SO_4_^2–^) that is present in the dye wastewater, and the formation of byproducts is shown in Scheme 1.

#### 4.2.1 Electrochemical Reduction of Indigo

Electrochemical approaches,^50^ hydroxyacetone,^15^ α-hydroxycarbonyls,^57^ bacterial reduction,^58^ fruit extracts,^59^ and glucose^60^ have all been offered as environmentally friendly alternatives for indigo reduction. In the direct electrochemical dyeing process, the indigo dyes are reduced directly; nevertheless, a small amount of conventional reducing agent is required to reduce the dyes to establish the chemical reaction; once the reaction has begun, the electrochemical reaction may be continued. Alternative electrochemical approaches for the reduction process may be the best solution to boost the eco-efficiency of current dyeing procedures for indigo dyeing on denim.^61,62^ It minimizes a number of chemicals used in the reduction process.^63^ The use of an ultrasonic system to perform electrochemical reduction on indigo dyes increases the water solubility followed by the dyeing efficiency, ensuring the environmentally friendly dyeing of denim under an aqueous system.^64,65^ The indigo dye particle size is reduced due to the ultrasonic atmosphere, and it is increasing the water solubility. This is a green technology that has numerous advantages over traditional (chemical) reduction techniques, including energy efficiency and the absence of chemicals (Na_2_S_2_O_4_ and NaOH). Electrochemical reduction for indigo minimizes chemical use; on the other hand, it consumes higher energy, and high surface area electrodes must be addressed to achieve complete sustainability in the indigo reduction.

In this context, hydroxyacetone is considered a green reducing agent for the reduction of synthetic indigo, and it provides a reduction potential of up to −810 mV vs Ag/AgCl/3 M KCl. Usually the indigo required a redox potential of >−700 mV vs Ag/AgCl/3 M KCl.^66^ The primary benefit of hydroxyacetone is that it helps bring down the significantly high levels of COD; as a result, the dyeing effluents do not contain any residual sulfites, sulfide, hydroxide, or strong alkalis. This reducing agent could not be brought to market because of the high cost of producing it. Because of its exorbitant price, commercialization of this reducing agent is not feasible. Glucose is a type of polysaccharide that can be utilized in the reduction of indigo^67^ and sulfur dyes.^54^ Glucose is considered nontoxic and biodegradable. Perhaps the most common application for glucose is to reduce sulfur dyes. Glucose-based reduction yields very good results in closed form (i.e., continuous dyeing machines). Perhaps, the strong alkaline bath and higher temperature were required to achieve the maximum reduction of indigo dyes (i.e., redox potential from −650 to 700 mV); other polysaccharides like hexose, fructose, galactose, lactose, and maltose were studied in a similar approach, but fructose provides better results.^54^

Sulfur dyes are often used to dye denim to produce a darker appearance. Similar to indigo, sulfur dyes are also not water soluble. Sodium sulfide (i.e., a reducing agent) is used to make the sulfur dyes water soluble. The sodium sulfide used in the sulfur dyeing process has generated considerable environmental problems. Sulfide is able to discharge hydrogen sulfide, and it is considered a harmful material to living organisms and their health. As a result, nonsulfide-based reducing agents, such as glucose and fructose, are of great interest. These polysaccharides are available abundantly and are is considered biomaterials. The use of natural indigo in combination with naturally occurring reducing agents including glucose, fructose, and ferrous sulfate and alkali derived naturally substances such as wood ash and limestone are considered organic denim and environmentally friendly dyeing techniques. Nevertheless, this organic denim is too expensive for mass production.

#### 4.2.2 Supercritical CO_2_ Dyeing on Indigo

**Figure 7:**
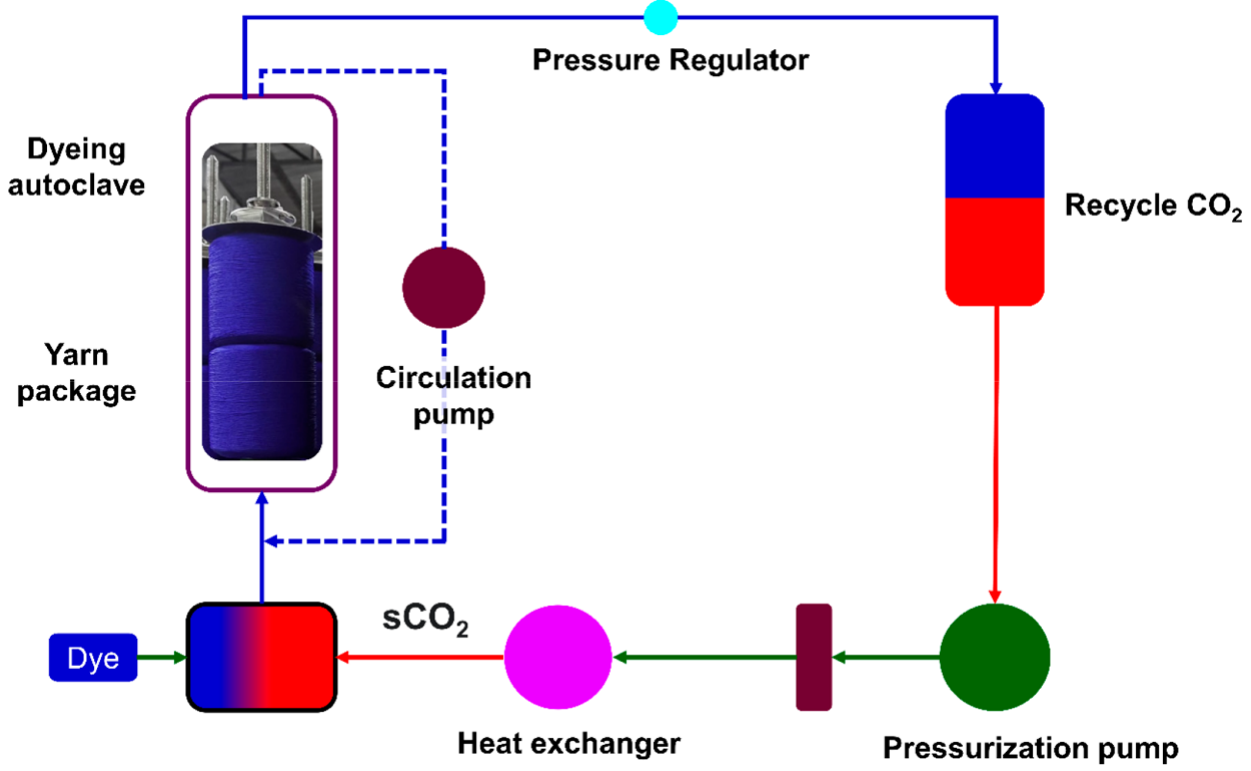
Supercritical CO_2_ dyeing set up for yarn dyeing.

Due to the benefit of using a clean solvent that can be readily collected and separated from the dye bath at end of the process, supercritical dyeing might be an attractive alternative to conventional dyeing. Supercritical fluids have been employed in a variety of applications for the last three decades, ranging from traditional extraction to advanced manufacturing.^68^ Supercritical dyeing can be an interesting alternative to traditional dyeing due to the advantage of the use of a clean solvent that can be easily recovered and separated from the excess dye at the end of the process. The use of supercritical CO_2_ in PET dyeing reduces the cost of effluent treatment.^69,70^ Indigo dyes are often water insoluble; however, they can be reduced when a supercritical CO_2_ (sCO_2_) fluid is used in the dyeing process^71,72^ (see Figure 7).

The addition of an organic solvent increases the solubility property of indigo colors by creating a strongly reducing environment.^73^ DyeCoo recently released beam dyeing equipment that uses supercritical carbon dioxide technology.^74^ The CO_2_ is cleaned after the dyeing process, and 95% of it is recycled back into the machine to be utilized. This technology saves not only water but also chemicals as well as speeds up the dyeing process by 40%, and reduces energy consumption by 60%. Fabrics do not need to be dried because it is a waterless technique, which saves money on the drying process.^75,76^

#### 4.2.3 Digital Spray

Digital spray is spraying the exact amount of dyestuff and finishing chemicals required directly onto the fabric with the help of nozzles. The dyeing process is incredibly efficient since it is digitally controlled. As a consequence, it utilizes far less water, dyestuff, and other chemicals than the conventional process, resulting in much lower effluent discharge than the conventional process. Furthermore, the spray dyeing procedure is different from digital printing in that it designs on the surface of the fabric, whereas spray dyeing primarily dyes the fabrics in solid colors with deep penetration. As a result, digital printing frequently necessitates the use of expensive specialty inks, whereas spray dyeing may use conventional dyes. Alchemie has created a digitally enabled spray dyeing technique combined with their unique airflow technology, which allows the dyes to be thoroughly penetrated inside the fiber structure. The infrared radiation heat is used in the fixation process to fix the dyes on the fabric. The method is compatible with conventional dyes and suits cotton and polyester materials. This method, according to Alchemie, reduces the carbon footprint of dyeing by over 85%, eliminates effluent from textile dyeing, and allows dyeing operations to be colocated with garment production in water-scarce areas. Overall, the technique cuts operational expenses by more than one-half.^77^

Imogo^78^ has created a digital spray for the dyeing and finishing process. To achieve deep penetration, digital spray dyeing uses the capillary forces in the materials with the help of absorption of natural fibers (i.e., in-built absorption properties of natural fibers). Traditional techniques of fixing, such as cold batching, are used after the dyeing process. Commercial dyes are compatible with the dyeing process, and the company is now concentrating on cellulosic materials, which are two advantages of this approach.

#### 4.2.4 Microbial-Assisted Dyeing

The use of microbes for dyeing has no harmful or carcinogenic-causing properties. To do this, David et al.^79^ worked on engineering microbes using DNA to turn agricultural byproducts into dyes. Once the appropriate colorant’s DNA code has been inserted, the organisms themselves may be grown or fermented because natural reproduction is a quick and effective process. To use less water and chemicals, they cultivated bacteria and mimicked their naturally existing color in an innovative method that is inspired by nature.

#### 4.2.5 Foam Dyeing

**Figure 8:**
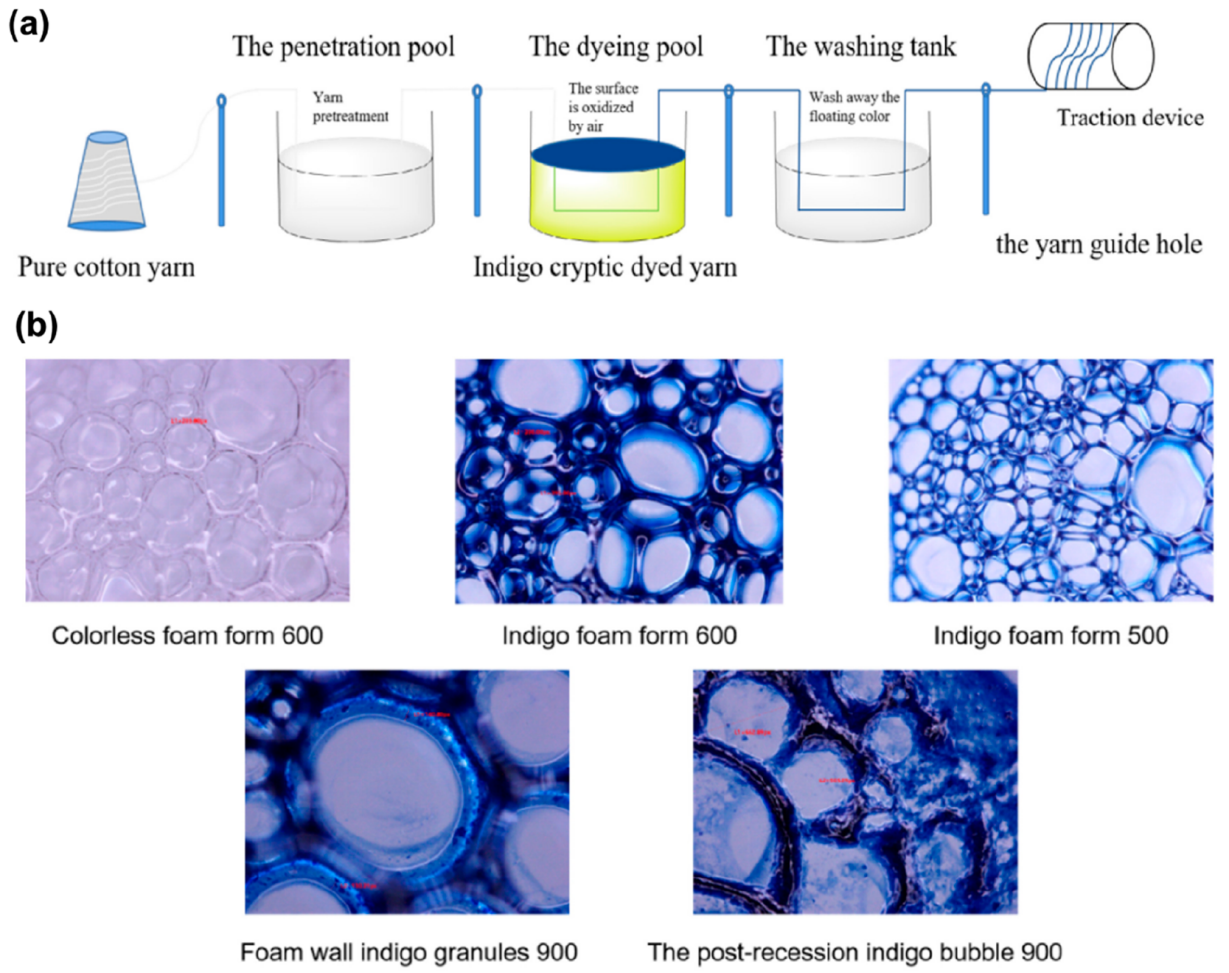
Process sequence for foam dyeing on yarn with indigo dyes (a); different bubble system generation for foam dyeing (b). Adapted with permission from ref (80). Copyright 2022 Elsevier.

Foam dyeing is a technique that uses foam as the dyeing medium (i.e., the dye is carried by the foaming agent). The foaming agent and stabilizer are mixed in an aqueous solution at a high speed to form the foams. Zhu et al.^80^ investigated the use of indigo dyes in foam dyeing on cellulose fibers. After stirring at high speed for a long period at a specific temperature, the dye with foam system (i.e., foam indigo) was created. Then, the cotton yarn was foamed, the assembly line colors used it, and it was dried immediately around the cylinder; the foam dyeing line is well described in Figure 8.

Foam dyeing with a low liquid rate reduces wastewater treatment issues, achieves low energy consumption during the drying process, and produces superior dyeing results. Furthermore, the foaming, which includes the dye particle, may be reused after defoaming and foamed by stirring again to lower the process cost. Foam technology has the potential to save 40% of the water used and 60% of the energy used.^81^ In comparison to the current approach, foam technology requires less water, uses less energy, and discharges less effluent. It can reduce liquid flow by at least 40% and save water by 40%, energy by 50%, and dyes and chemicals by 20%.^82^ Foam dyeing is a water-saving, sustainable dyeing process used in many textile industries around the world. The inability to utilize air to make the indigo foam medium, however, has hindered its usage for indigo coloring denim yarns. To get around this limitation, Zhu et al.^80^ developed a design that keeps the environment oxygen free until the yarns are prepared to be exposed to either natural sunlight or an oxidation chamber.

#### 4.2.6 Dope Dyeing

Dope dyeing is a production technique to color man-made fibers including polyester, polyamide, lyocell, viscose, and acrylic. In the dope dyeing method, the colorants are incorporated into the polymer solution before the extrusion of the fibers. In comparison to conventionally dyed textiles, color fastness qualities are improved in dope-dyed textiles as the colorants are incorporated into the polymeric structure. Lenzing introduced the one-step spin-dyeing process on Tencel and Modal fibers.^83^ While utilizing fewer resources, saving chemicals and electricity, less wastewater discharged, and no use of thermal energy, this technology provides better color fastness compared to conventional indigo dyeing. Dope dyeing is a one-step process that involves the production of fibers, and dyeing takes place. Compared to traditional dyeing, dope dyeing consumes 80% less water, more than 20% less dyestuff, 80% less chemicals, and 7% less power.

The Colorbox was developed by Jeanologia, which is a full line of state-of-the-art garment dyeing machinery.^84^ Utilizing Colorbox technology, Levi’s reduced the amount of water used to finish denim by 1.3 L, saving between 30% and 90%. According to Colorbox, it may lower the usage of salt by 76%, water by 60%, energy by 45%, and chemicals by 60%.^84^

### 4.3 Enzymatic Desizing

Enzymes are engaged in several processes, the most common of which is hydrolysis. Other reactions involving enzymes include oxidation, reduction, coagulation, and decomposition. The enzyme is frequently used in denim processing since it is biodegradable. The behavior of an enzyme with a single substrate is explained by the lock–key concept. The different enzymes and their reactions are shown in Table 2 for the numerous enzymes employed in the textile and denim industries. Desizing is a technique that can remove the starch as well as any other ingredients that were added during the sizing process. Traditional desizing has limitations and drawbacks that are overcome by enzymatic desizing. α-Amylase can remove insoluble starches from denim by simple hydrolysis techniques.

**Table 2:** Classification of the Main Industrial Enzymes and Their Examples and Reactions

enzyme class	types of reactions catalyzed	enzymes
hydrolases	hydrolysis of molecules and degradation in some cases	cellulase, protease, amylase, lipase
lyases	nonhydrolytic cleavage of degradation of the molecule	fumarase
transferases	transfer a group from one molecule to another	transaminase
oxidoreductases	oxidation or reduction of molecules	laccases
isomerases	conversion of one isomer to another	glucosephoshate, isomerase
ligases	joining of two molecules with adenosine triphosphate (atp)	glutamine synthetase

### 4.4 Finishing Techniques

#### 4.4.1 Sustainable Wash-down Effects

The use of a cellulase treatment in denim fabrics is an important step in producing the stonewashed (i.e., worn-out look) appearance of denim. Cellulase treatment of denim is an environmentally friendly way of improving the property of the fabrics. Cellulase enzyme is used in a procedure known as “bio stone washing”, which speeds up the abrasion process. The washing process for denim garments can be carried out under more gentle conditions, and cellulase can replace pumice stones as well as other agents that are toxic and harmful. Since few grams of cellulase replace the kilograms of pumice stones, enzymes reduce the fabric damage. The faster reaction time of cellulase is another factor that can contribute to enhanced productivity. Sediment-free wastewater reduces the effluent load for the treatment plant. There are various classes that cellulase including the endoglucanases (EGs) attack soluble cellulose. Cellobiohydrolases (CBHs) convert crystalline cellulose to water-soluble glucose molecules. Cellobiose-attacking cellobiose and β-glucanases break down big chains. Both acid and neutral cellulases are used for biowashing; however, acid cellulase is more aggressive and attacks cellulose’s 1,4-glycoside linkages more rapidly. As a result, it produced a wide range of abrasion effects on denim and required less processing time. However, it causes more fabric damage and back staining than other methods.

#### 4.4.2 Biopolishing

Biopolishing is a process for improving the physical appearance of denim fabric. Denim is often made from cotton, which generates microfibers owing to mechanical action during the manufacturing process; this is also caused by the presence of short fibers. Although the generation of fuzz by these microfibers is acceptable in some applications, fuzz generates a huge quantity of microfibers during domestic washing as it is considered a series of environmental threats.^25^ Therefore, this technique removes these microfibers from the denim fabric and considerably improves the appearance, smoothness, and brightness of the fabric.

Recently, lyocell has been the primary material used in the production of denim. The fibrillation properties of lyocell denim fabric may contribute to the formation of pilling on the surface, thereby diminishing the overall appearance of the denim fabric.^85,86^ Numerous techniques, including alkali^86−90^ and enzyme treatment, could be used to reduce fibrillation on lyocell fibers. The enzymatic approach provides a better appearance for the lyocell denim fabric. Since the untreated fabric has a greater percentage of protruding fibers, investigations by Nisha et al.^91^ of the biopolishing of cotton fabrics confirmed that the protruding fibers on the surface of the fabric are removed by enzymatic treatment. In contrast, immobilized enzymes have a higher removal efficiency than regular enzymes. Additionally, it minimizes the loss of strength and weight of the fabric.

### 4.5 Sustainable Bleaching

Bleaching is the process to remove or decolorize the indigo from the denim fabric surface. Typically, a strong oxidative bleaching agent like sodium hypochlorite (NaOCl), KMnO_4_, and H_2_O_2_ is used, and the bleaching can be done with or without the addition of stones. The denim bleaching process in the industry often involves the use of NaOCl. Notably, the NaOCl treatment causes the fabric to yellow since it contains chlorine, which is also responsible for the mechanical properties of denim fabric. However, the NaOCl treatment on denim has the potential to release hypochlorous acid, and residual chlorine is hazardous to the environment since it threatens living beings and affects the ecosystem. As a result, it affects the lungs and creates many other acute respiratory distress syndromes. Moreover, NaOCl is a highly irritating chemical that has the potential to cause significant chemical burns to the people who work with it. In addition, strong reducing agents (i.e., thiosulfate or sodium metabisulfite) are utilized to remove any residual chlorine from the denim fabric (i.e., antichlor process). The utilization of reducing agents results in the production of foul-smelling gas and SO_2_, both of which are detrimental to the environment. Since chlorine is toxic, the hypochlorite bleaching process is extremely unpleasant as it induces chlorine and salts to be released in large quantities and raises the levels of BOD and COD. In these situations, industry and researchers are attempting to replace traditional technologies with more environmentally friendly alternatives. The denim bleaching industry has recently become more familiar with ozone, electrochemical, plasma, laser, and advanced oxidation process (AOP) procedures as ecologically acceptable alternatives to decrease the use of chemicals, time, energy, and water. Table 3 consolidates the various discoloration techniques on the denim fabric with the advantages and disadvantages of each process.

**Table 3:** Various Sustainable Decoloration Methods for Denim Processing

discoloration techniques	advantages	disadvantages	refs
ozone discoloration	deep decoloration, eco-friendly, simple, and efficient	possible strength loss, yellowing tendency, investment is very high	(92−95)
biowashing	simple process, eco-friendly, less damage to the denim (except acid cellulase), and possibility to recycle and reuse the enzymes	back-staining possibilities during washing	(96−100)
electrochemical process	energy efficient, faster, and eco-friendly	yet to be commercialized	(49,50,101−103)
plasma processing	faster decolorations required less energy, no discharging of waste	technical challenge, difficult for repeatability	(95,104−107)
laser processing	faster decolorations required less energy, no discharging of waste	technical challenge, difficult for repeatability	(108−112)
aop	no damage on the fabric, eco-friendly, corrosion and yellowing free	yet to be commercialized	(113−115)

#### 4.5.1 Ozone Fading

One of the ecological alternatives for denim bleaching is ozone (O_3_) treatment. It is a powerful oxidizing agent chemically, which causes it to oxidize more quickly than other oxidizing agents. Ozone may damage living cells if inhaled; thus, caution should be taken when applying it to denim fabric. When denim is exposed to ozone, the indigo on the surface transforms into isatin and anthranilic acid, causing it to fade or start to yellow; their chemical reaction is shown in Figure S2. The biggest disadvantage of ozone is that it costs more to operate than traditional hypochlorite bleaching. The advantages of ozone fading include faster treatment, a smaller number of processes, and saving energy, water, and the environment.

The carboxylate carbon nanotubes (CNTs-COOH) made the ozone treatment on indigo more effective.^116^ When compared to the traditional method, the overall results show that ozonation in the presence of CNTs-COOH provides a considerable influence on the decolorization, which results in an improved level of brightness. The authors made an effort to evaluate a variety of catalysts and discovered that carboxylic functional groups can act as catalysts for the ozonation process. According to molecular orbital theory, the position and orbital energy of an attacking chemical are crucial factors in determining its level of reactivity. Throughout this process the hydroxyl radical of the double bond that is present in indigo pigments could be attacked.

Jeanologia is the Spanish company that developed the ozone fading instrument “G2 Ozone”.^117^ In addition to other environmental benefits, such as cleaning any residual indigo redeposition and controlling the cast of the fabric; this technique is the most advanced and eco-efficient ozone technology. In addition, it produces zero waste and achieves considerable reductions in water use by 65%, energy use by 20%, and chemical use by 80%.^117^

##### IoT, Artificial Intelligence in Ozone Fading

As raw denim is colored with natural indigo dyes or other combinations of dyes like sulfur, reactive, or synthetic indigo dyes, a few critical parameters should be addressed to produce successful ozone bleaching on denim. Since ozone bleaching works efficiently on denim with natural or synthetic indigo dyes alone, it will not work on sulfur-colored denim to produce a fresh look (i.e., a similar look obtained from indigo-dyed denim). In addition, the ozone accelerates its reaction in acidic conditions, and with the presence of moisture on indigo-colored denim, the outputs are faster and uneven.

On this occasion, moisture plays a vital role in the bleaching reaction, making it so optimum moisture levels in the machines can be achieved with the help of the Internet of things (IoT) and artificial intelligence (AI). Few start-ups come up with this concept to embed artificial intelligence, IoT, and machine learning into the new machinery to ensure the best quality of the process with improving sustainability.^118^ Existing machines with programmable logic controller (PLC) panels will not support the level of sustainability in terms of health concerns since the IoT and artificial intelligence in ozone management will protect humans from long-term ozone exposure. The support of digital infrastructure will be the appropriate solution to protect the health and hazards of ozone chemistry. This technique offers potential not just for ozone bleaching but also for chemical handling in the denim industries owing to Industry 5.0 (5IR), IoT, and AI. IoT, AI sensors can be used during the denim bleaching process with the denim parameters including the moisture level and the machine parameters like ozone concentration (i.e., available ppm) to determine the optimum bleaching process with the help of machine learning. It also helps to monitor the ozone concentration and moisture to protect humans from exposure to ozone gas.

#### 4.5.2 E-Flow

Jeanologia developed and patented a novel technology known as e-flow based on nanobubbles. By using nanobubbles of air instead of water, one can replace traditional abrasion processes while also providing superior performance for the fabric without affecting its mechanical and physical properties. An electroflow reactor receives air from the outer air, which is then subjected to electromechanical shock, which results in the formation of nanobubbles and a flow of moist air (Figure S3). It is lowering the expense of the application, decreasing 95% of the water, 90% of the chemicals, and 40% of the energy that is consumed.^119^

#### 4.5.3 Enzymatic Bleaching

**Figure 9:**
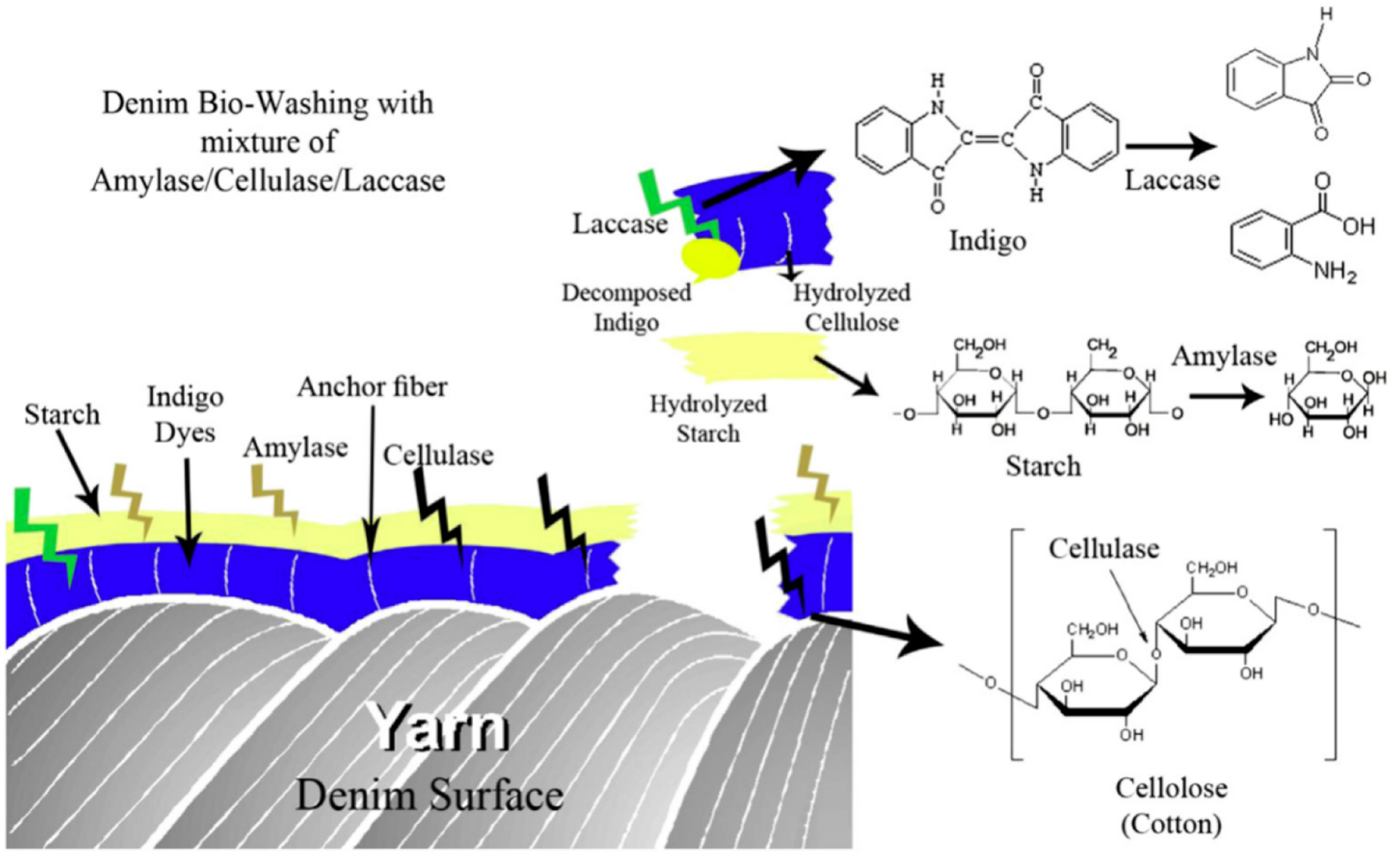
Schematic mechanism of denim biowashing. Adapted with permission from ref (123). Copyright 2013 Elsevier.

The use of laccase has increased in the denim industry, particularly in the bleaching process where it excels. A sustainable replacement for traditional NaOCl bleaching is lacase-based bleaching. However, the laccase cannot be used on denim clothing that contains Lycra. The laccase is typically a “redo” type of enzyme with molecular oxygen serving as an electron acceptor. Under an aqueous environment, lacasse oxidizes and attacks the mediator, which produces free radicals^120−122^ (Table 4). As a result, the free radicals continue to destroy indigo to produce isatin and anthranillic acid. Figure 9 depicts the bleaching method using several enzymes. Table 4 describes the various research on enzymatic bleaching.

**Table 4:** Various Research on Enzymatic Bleaching on Fabric

type of enzyme	work carried out	refs
laccase	laccase and peroxide combined bleaching	(124)
	ultrasonic-assisted laccase bleaching	(125,126)
	whiteness improvement over conventional bleaching	(127)
	improvement of whiteness on combined bleaching with ultrasonic and laccase	(128,129)
glucose-oxidase	bleaching of cotton with glucose	(130−132)
	combined desizing, bleaching	(133)
	improved whiteness on bleaching	(134)

##### Advantages of Laccase-Based Bleaching of Denim

Laccase works more quickly than traditional bleaching techniques. When compared to acid cellulase, it adds more efficiency while using less enzyme input. The primary advantages of bleaching with laccase are

- the least amount of strength loss (i.e., if the parameters are optimized),
- more rapid decoloration, which reduced water use,
- an environmentally friendly replacement for traditional chlorite bleaching.

##### Cold Enzyme DeniLite

The novel enzymes “DeniLite” created by Novozymes can work at room temperature.^135^ It was created for the bleaching of textiles including denim fabrics. This enzyme is based on peroxidase, which unlike laccase enzyme carries out the bleaching process without the need for oxygen (often from water, air, and fabric surfaces). In contrast to laccase enzymes, which need oxygen to carry out the bleaching process (often from water, air, and fabric surfaces), DeniLite belongs to the peroxidase family.

#### 4.5.4 Water Jet Fading

In the process of water jet fading, high-pressure water is used to abrade the surface of denim to give it a more worn-in look. The process involves exposing either one or both sides of the garment to hydro-jet nozzles. The quantity of fluid dynamics that is applied to the fabric as well as the type of dye that is used in the fabric all affect the degree of fading, the clarity of the patterns, and the softness of the cloth that is produced. This method does not involve the use of any chemicals, agents, or other additives, so there is no risk of pollution being caused by it. This can be a very cost-effective and ecologically responsible method of processing denim if it is equipped with a water recycling system.^136^

#### 4.5.5 Laser Fading

Laser technology is not new, and the denim industry, semiconductors, and medical industry have been using it for a long time; however, optimizing the laser power plays a vital role to improve productivity and fulfill expectations. Lasers have high potential in the denim industry to bring a lot more sustainable value to the clothing and fashion industry by moving this approach for development of laser systems. The key aspects of laser are as follows:

- color fading efficiency (i.e., natural color fading),
- software algorithm,
- burning mechanism,
- mechanical moment of the machine.

The laser produces light energy and intensity, and power can be controlled.^137−139^ This is one of the most environmentally friendly methods for dry-finishing denim to produce stone wash or sandblasting effects.^111^ To produce worn-out effects, a laser induces the thermal degradation of indigo. The fading efficiency depends on the laser wavelength, power density, exposure time, and beam width.^140^ Using laser fading offers numerous benefits:

- a process for fading denim without water,
- an environmentally responsible method,
- a reduction in process costs.

**Figure 10:**
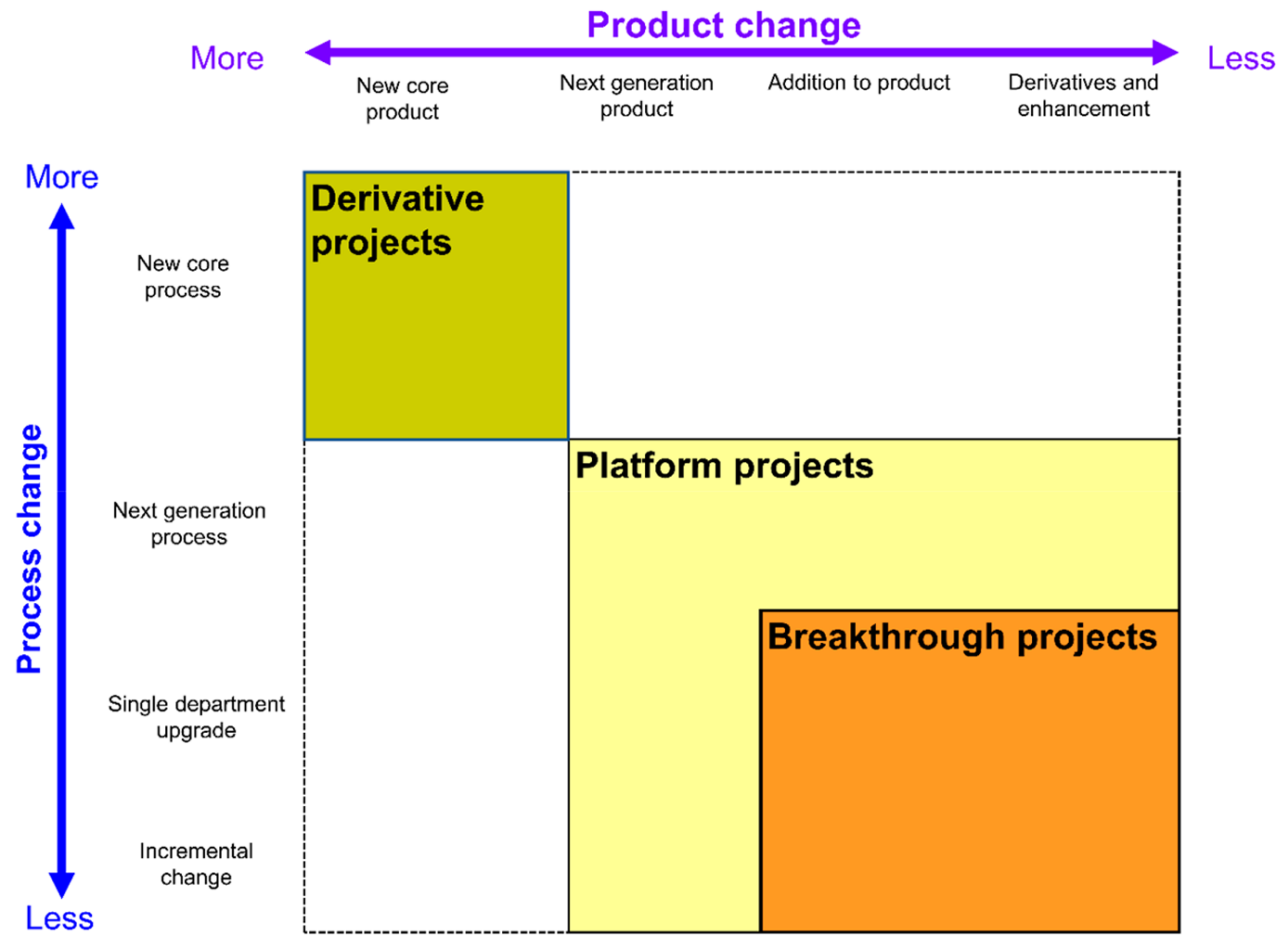
Project planning for the development of laser fading on denim.

The raw usage of lasers exists compromising the final aesthetic of the product owing to a need for sustainability (i.e., companies pushing, consumers expecting), but the true potential of this technology has yet to be explored and handled. To date, a percentage of laser-faded denim required human touch up (i.e., more energy, air pollution) to be commercially sellable. Nonetheless, the laser machine requires a large investment, but it will not match the customer’s expectations of real sustainability and aesthetics. Lower power lasers are employed in denim fading; lasers require a vacuum environment to preserve laser quality and the lifetime of the laser tubes, as the laser strength is continually decreasing owing to CO_2_ interruption in laser tubes. If the laser tube is maintained in a vacuum condition in everyday operation, it increases the lifetime of the laser. Most of the laser machines are working based on software algorithms in terms of design software and its connection to control the laser intensity (epi/dpi), but this approach only helps us to get a certain level of benefit. In this case, using a lens in the laser machine, like a DSLR shutter camera mechanism, to optimize the intensity through a filtering process can provide a far more natural look on the denim than the present laser technologies in the denim industry. As a result, the laser manufacturer should sync the laser algorithm, angle of garment location, and shuttering lens for the laser to increase fading quality and decrease or eliminate the requirement for manual touch up for laser-faded denim. Since Metaverse is emerging and 3D designs are the channel of choice for the customer (hyper-personalized), as of now, the laser machine and its algorithm can communicate with the 2D designs from Photoshop or Illustrator. Therefore, the current laser manufacturers developing their machine algorithm to communicate the 3D designs made by individual customers will help to produce demand-based denim to reduce the wastage and landfill of denim garments. The effectiveness of laser fading on the denim product development capacity ultimately depends on the development and optimization of the laser machine. This will increase productivity, in this case, there are few development initiatives in the laser fading on denim (Figure 10).

- Derivative project in laser: which improves the software algorithm and design illustrations—marking the position by sensors and a camera to enhance floor machines.
- Platform projects: improve the mechanical action of the garment placed in a location. Adjust the degree of the moment to be angled for laser burning.
- Breakthrough projects: using IoT and AI cameras and algorithm synchronization to convert 3D models or images directly in a laser system to eliminate manual designs to connect the multiple dots is the real solution for sustainability.

Venkataraman^93^ investigated the fading impact of denim using a laser and carbon dioxide and found that the applied laser power changed the brightness of the treated denim fabric. Due to the fading of the colors, denim fabric’s brightness has decreased as laser power has increased. Additionally, the provided laser power affects the color of the fabric’s surface. Chi-wai Kan^141^ investigated how laser treatment affected denim fading and compared the results to stone washing, resulting in the laser treatment saving water and time of processing. Conventional stone washing takes seven stages, and the laser only needs six. The stone-washing method required three rinses, while the laser only needs two. Stone washing took 45–60 min at a temperature of 55–60 °C. However, the laser fading was accomplished in 3 min at room temperature. Consequently, this procedure uses less time and energy and produces less effluent, making it a sustainable one. Jeanologia developed the denim-washing system known as laser pro washing which is 40% faster than other types of laser technology.^142^ As a result, it is reducing energy costs and makes the industry sustainable.

#### 4.5.6 Photocatalytic Discoloration of Denim

Photocatalytic decoloration is one of the most widely explored topics in the field of wastewater treatment due to its low-cost process. This technique, which is a part of the advanced oxidation process (AOP), assures those different dyestuffs will degrade while the wastewater is being treated. Izadyar et al.^143^ used AOP with H_2_O_2_/UV to research the photocatalytic discoloration of denim. The denim fabric was first treated for 5 min with a 30% H_2_O_2_ solution, washed, and then exposed to UV radiation for a different duration. The sample after treatment demonstrates that higher reflectance does not affect the color strength values. They concluded that this method holds great promise because it is more efficient and sustainable. In general, the application of AOP fading to denim helps increase the effectiveness of oxidation, which ultimately leads to a decrease in COD levels in wastewater treatment.

#### 4.5.7 Plasma Associated with Denim Processing

Over the last few decades, plasma technology has been applied to the process of treating textiles since the process is simple. The term “plasma” refers to a partially ionized gas that is made up of both positive and negative ions, electrons, neutrals, excited molecules, photons, and ultraviolet light. Other significant benefits of plasma processing over the conventional wet-chemical processing of textiles include the following: liquid-free and environmentally friendly dry operation; single-step faster operation; less chemical requirement; cost effectiveness in terms of processing time and temperature. Therefore, plasma processing is considered a sustainable approach to changing the surface properties of polymers and textiles.^144−146^ It is well-known fact that the ultimate results of the plasma-related process rely on the kind of gas, the processing time, the pressure, and the discharge power.^144,147−149^ Ghoranneviss et al.^150^ evaluated the fading effects of denim fabric using Ar and O_2_ in the low-temperature plasma (LTP) process with various exposure times; it was discovered that Ar-treated samples had lower K/S values when tested at the same frequency and for the same amount of time. Furthermore, the indigo-dyed denim fabric undergoes LTP and corona treatment, both of which are linked to the generation of reactive molecules and radicals in oxygen-containing gas mixtures, which in turn causes the indigo dyes to oxidize and provide the desired faded effect.^151^

### 4.6 Developments in Enzyme Application on Denim Processing

Enzymes are a green alternative and a type of bioprocess that not only results in a cleaner process but also saves time and energy, both of which are indirectly correlated with the carbon credits owed to the denim industry. However, there are some limitations to using enzymes since they are susceptible to temperature and pH; hence, current research has focused on enzymes with improved activity, which also includes circumstances with higher temperature and pH. It can be improved by immobilization of the enzyme and a combined enzymatic process.

Since its first discovery, there has been an incredible amount of development taking place in enzyme technology. The past two decades have seen the emergence of biocatalysis as a major technology to address the expanding need for environmentally friendly and sustainable textile processing. Immobilization of the enzyme is one method that can be utilized to improve the enzyme’s characteristics. For instance, it helps to increase the enzyme’s resistance to changes in temperature or pH.^152−158^ To immobilize the commercial cellulase for biopolishing treatments,^159^ methanol was employed in conjunction with ion-exchange resin and epoxy resin. Epoxy resin, on the other hand, exhibits a higher percentage of cellulase immobilization than ion-exchange resin does. According to the author’s observations, the biopolishing result on cotton fabric treated with immobilized cellulase was effective for six cycles in a row, resulting in less strength loss when compared to cotton treated with cellulase.^159^ In another study, commercial cellulase that had been bound to zirconyl chloride made pumice particles immobilization for biostone effects on denim, the results show that the immobilized enzyme fades the denim surface more efficiently.^160^ Because of this, immobilized cellulase has a lot of potential in the biowashing/stoning of denim fabrics.

Ultrasonic (US) energy was first used in enzymatic desizing using amylase enzyme by Wang et al.;^125^ the desizing efficiency was increased with the introduction of US energy. Utilizing these methods has the benefit of increased productivity in a short amount of time. The findings were consistent across several studies.^161−163^ The structure of the protein is altered because of ultrasonic cavitation, which increases the activity of the enzyme. Combinations of different enzymes (amylase/cellulase/laccase enzymatic treatment) in the same bath were studied, and the overall results were observed to reduce water usage, energy consumption, and other resources.^123^

### 4.7 Reuse of Recovery of Indigo

Reusing chemicals and dyes that have been recovered from industrial waste reduces the environmental impact. For instance, numerous methods, including neutralization, filtration, leaching, evaporation, and electrodialysis (ED), are used to remove caustic soda from industrial effluent. As was mentioned in the prior part, indigo dyes are notoriously challenging to decompose, which results in several negative effects on the ecological system. Dennis^164^ developed a method that was both efficient and cost effective for recovering indigo from denim dyeing effluent by simply adsorbing it with palygorskite clay. In another work, indigo was recovered by using four different ultrafiltration membranes.^165^ Later, the recovered indigo was used to dye the fabric, and the results of color difference, color strength, and fastness properties showed that indigo acted like virgin indigo dyes. Jeanologia developed a closed loop system (H_2_Zero) for denim processing that reuses the resources, and that leads to reduced water and energy consumption and zero discharge.

### 4.8 Microfiber/Microplastic Reduction

The rise in the global population is directly responsible for the acceleration in the production as well as the consumption of textile products. Microplastic pollution is predicted to become increasingly widespread as the human population continues to expand and as people continue to use more synthetic materials. Textile microfibers have been found in marine sediments and organisms, posing a real threat to the environment.^25,166,167^ Denim is one of the most widely used types of outerwear all over the world. Unfortunately, denim is the largest contributor to the pollution caused by microfibers.^27^ Denim fabric that was manufactured with 97% PET and 3% Lycra was found to have an average of 2 300 000–4 900 000 microfibers per 1-kg wash load according to the findings of researchers; the release was reduced to 27.5% of microfibers for 50% PET and 50% cotton blended jeans.^27^ To protect against the damaging effects that microplastics can have, the formulation of mitigation strategies is urgently required. Sustainable finishing techniques used for denim can assist in dramatically cutting the formation of microplastic and microfibers that are produced by denim garments during domestic washing, and these techniques will demonstrate a substantial reduction in both microplastic and microfibers even before they reaches customers.

#### 4.8.1 Mechanical Finishing

**Figure 11:**
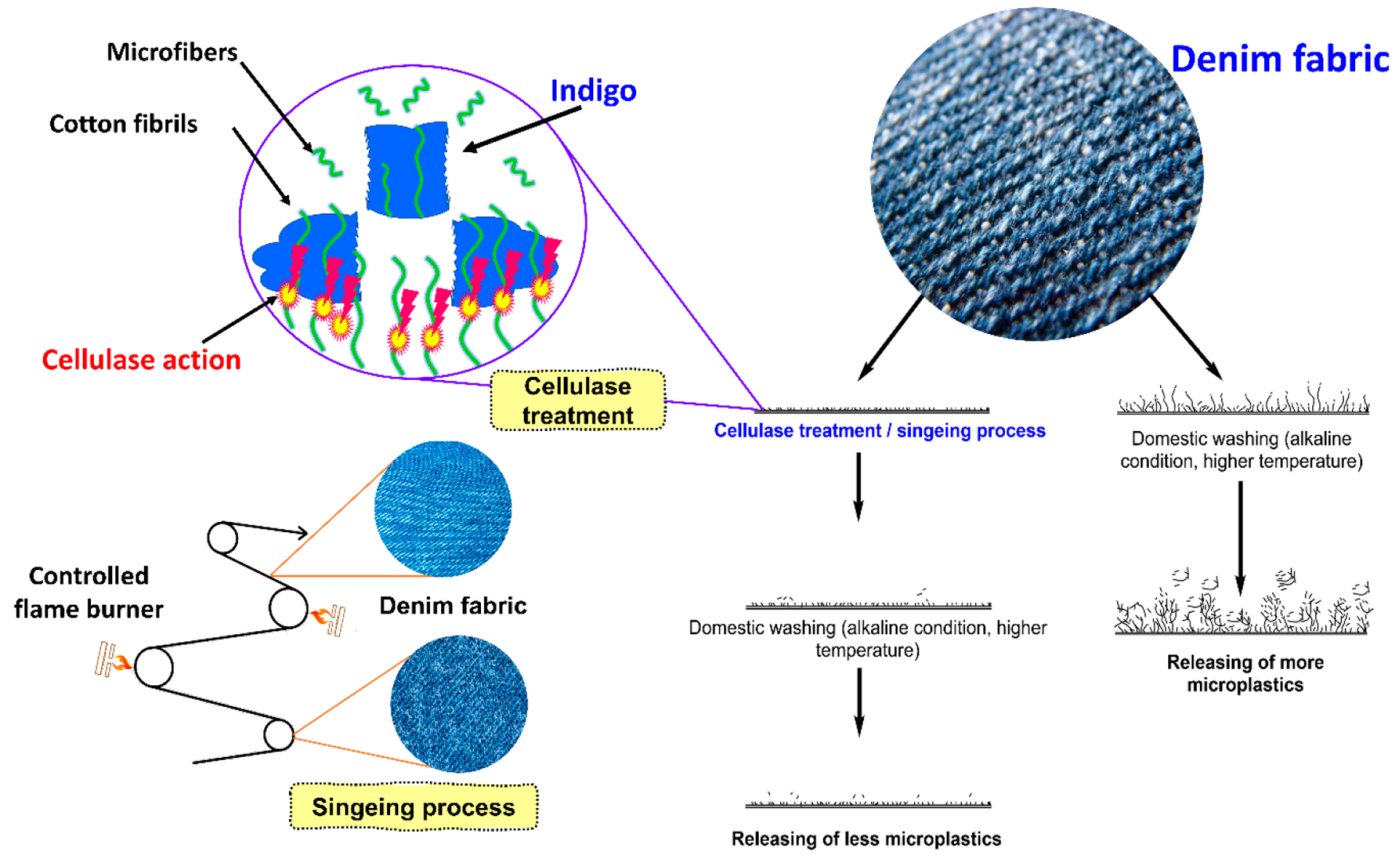
Mechanism for the microplastic generations from denim and their possible finishing techniques to reduce the microplastic generations.

Mechanical finishing can be considered a sustainable finishing technique on denim fabric as it will not use any chemicals or water and generates no waste. Singeing and calendaring are two examples of mechanical finishes that help reduce the release of microplastics. Singeing is a finishing process that is used to smooth the surface of the denim (Figure 11). This is accomplished by using a controlled flame to remove the microfibers that are on the surface of the fabric. The denim fabric is compressed during the calendaring process by passing between two or more rollers under carefully controlled conditions of time, temperature, and pressure.^25^ Shearing and brushing are examples of mechanical surface treatments that dramatically change the denim surface, hence enhancing its ability to increase the release of microplastics. To remove the indigo dyes from the surface of the denim garments, there are different finishing techniques, including stone washing, sandblasting, scraping, whiskering, tacking, grinding, and ripping cuts, utilized. All of these finishes open the structure of the yarn; as a result, they increase the number of microplastics/microfibers during the subsequent washing.

The link between the supply chain and the sustainable developments taking place around the ecosystem is an essential component of the growth of Industry 4.0. The information technology developments have shown significant revolutionary progress; however, the supply chain of products is disconnected as a result of a silo mentality of approaching products and sustainability. The era of the fourth industrial revolution (4IR) or Industry 4.0 was ushered in by the introduction of intelligent devices that make use of the wireless networking and cloud computing infrastructure. These developments have enabled the enormous possibility of interconnectedness between machines and machines as well as between humans and machines. The 4IR is the name given to the process of integrating this kind of network into an environment where production and operations are carried out.^168,169^

**Figure 12:**
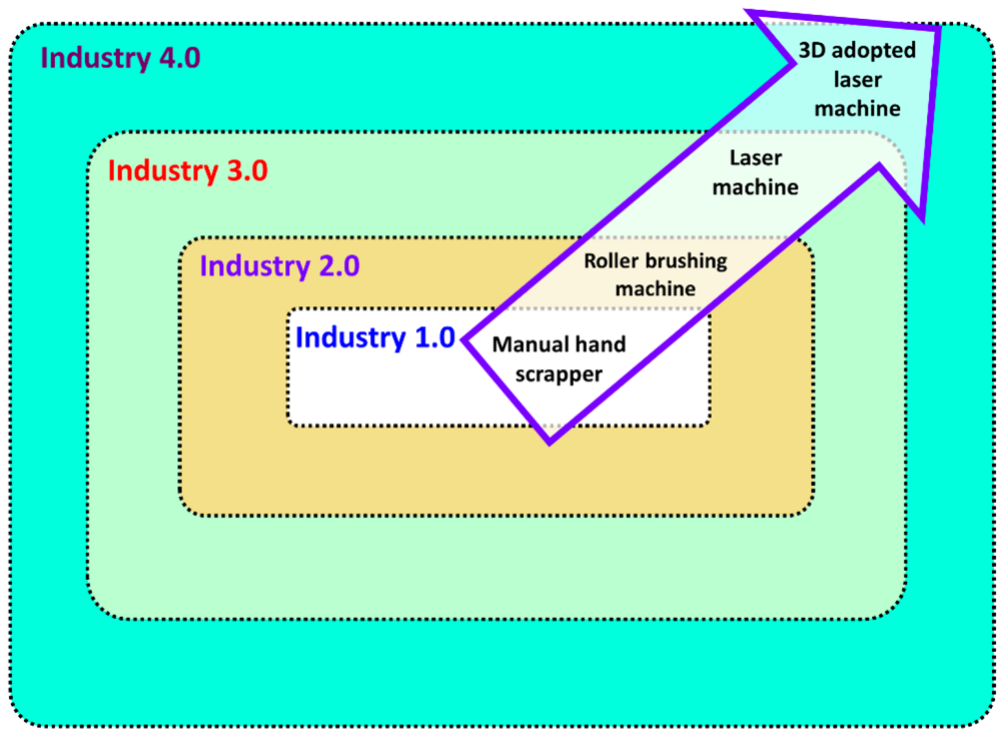
Industrial revolution in denim processing.

During the early stages of the industrial revolution, the denim industry relied heavily on a number of manual processes, such as manual hand scrapping, and it revolved around roller brushing machines. Later, the industry transitioned to using laser machines, and 4IR now provides the platforms necessary to use 3D-adopted laser machines (Figure 12). The additive manufacturing, big data analytics (data analytics, machine learning, artificial intelligence for data lacking), cloud computing (enterprise resource planning, SAP), cyber security (supply chain extension for the data feed), Internet of things (IoT traceable techniques including a radio frequency identification device (RFID), NFC built yarns or fabrics), collaborative robotics (machine to machine communications, 3D designs, avatars, digital twin), and visual computing are some of the layers that 4IR offers in the denim supply chain toward sustainable approaches. Indeed, 4IR and the technological improvements it entails are poised to have a huge impact on the businesses that make up the supply chain in order to bring about transparency. These new sustainable physical developments like machines, methods, and technology are all linked to the digital layers of IoT, and big data will bring resilient supply chain transparency and efficiency in the ecosystem, helping sustainability at a higher rate of growth compared to postera. In the processing of denim, it is necessary to conduct a competitive analysis in which each technology, such as a laser, bioenzyme, and spray indigo dying method, is evaluated in terms of price and sustainability. When, what, and how this technology is practically adopted and economically affordable also can be calculated using different measures (S-curve analysis, Pareto model, and Moore’s law) and implemented at the right time. Due to the obvious growing concern that global warming is causing in each and every nation and region, adopting a sustainable approach is no longer a choice.

To be more specific, it is proposed that 4IR be implemented in the denim supply chain in order to improve sustainability initiatives by means of improved design for the environment, cleaner production, controlled consumption of resources such as energy and raw materials, proactive maintenance, carbon-efficient logistics, and extension of product lifecycles through reuse, repair, recycling, and remanufacturing of products. Additionally, it ensures manufacturing with smart factories that are participating in initiatives to use renewable energy, efficiently allocate resources (i.e., specifically, products, materials, energy, etc.), and build a cross-linked value chain consisting of interconnected factories, products, and services across a variety of companies located in a variety of countries. In fact, sustainability activities in this day and age of 4IR have made significant strides forward to the implementation of practices such as remanufacturing green product design, green manufacturing methods, and green logistics.^170^

## 5 Conclusions

The modern denim industry is one of the biggest contributors to the textile-based global economy and essential to the economic and social growth of many developing countries. The industry of denim is expanding at a faster rate as a result of several factors, including urbanization, the westernization of lifestyles, rising fashion consciousness, and an increase in the desire for a more casual appearance. Unfortunately, the method of manufacturing denim has a negative impact on the environment due to the release of colored effluent, heavy metals, acids, alkalis, enzymes, and other pollutants. This review provides an overview of various environmentally friendly chemical processing techniques to produce sustainable denim.

- To overcome the issues, a startling amount of multidisciplinary advanced research is necessary with a particular focus on the synthesis of bioindigo employing genetic engineering and synthetic biology to modify the microbial synthesis of indigo as an effective alternative to synthetic indigo. Future trends in denim dyeing technology include the synthesis of bioindigo, waterless indigo dyeing using supercritical carbon dioxide, digital spray, and foam dyeing. Nevertheless, the synthesis of bioindigo has attractive market potential, since this synthesis requires less water, land, and energy than the plant-based indigo dyes.
- Digital spray, foam dyeing, dope dyeing, and sCO_2_ dyeing on denim offer promise for future sustainable denim due to their remarkable potential as alternatives to conventional dyeing techniques.
- The denim manufacturing process includes a step called desizing, which causes the generation of wastewater with a high degree of BOD. While some researchers have attempted to find ecologically friendly replacements for existing sizing materials (soy protein, chicken feather, hemp core, and cellulose ether), it is not viable to scale this up to an industrial level.
- In the process of finishing denim, enzymes may be used as a feasible alternative to traditional finishing methods (stone washing), conserving both water and energy. Now is the time to develop the immobilized enzymes to boost their reactivity and multifunctionality (i.e., combine processes), which will have more promising impacts on the denim sector.
- The use of ozone and laser-assisted bleaching on denim has shown remarkable potential due to the fact that it can be carried out at room temperature, requires significantly less time for processing, and, most importantly, is more environmentally friendly than conventional bleaching (i.e., chlorine bleaching).
- Sonication and plasma-assisted denim processing have previously been proven at the laboratory level, and there is a challenge in the designing of machinery on a bulk scale as it requires huge capital investment, which prevents them from being widely used.

## Supporting Information Available

Oxidation and reduction of indigo, ozone bleaching mechanisms, and e-flow process of denim (PDF)
