# Supplementary material for: Critical Review on Sustainability in Denim: A Step toward Sustainable Production and Consumption of Denim
Source: ACS Omega. 2023 Jan 13;8(5):4472–90. doi: 10.1021/acsomega.2c06374 (PMC9909807; doi:10.1021/acsomega.2c06374)
Supplement: Supplementary file 1 — ao2c06374_si_001.pdf [file ao2c06374_si_001.pdf]

Supporting Information (SI) for

Critical review on Sustainability in Denim: A step towards sustainable production and consumption of denim

Aravin Prince Periyasamy[1], Saravanan Periyasami[2]*

[1]Department of Bioproducts and Biosystems, School of Chemical Engineering, Aalto University, Espoo, 02150, Finland.
[2]R&D manager laundry operations, Thuan phuong co.,ltd (garments -embroideries), Vietnam.

[*]Corresponding author (APP): aravinprince.periyasamy@aalto.fi) &
(aravinprincep@gmail.com)

Number of Pages: 2

Number of figures: 3

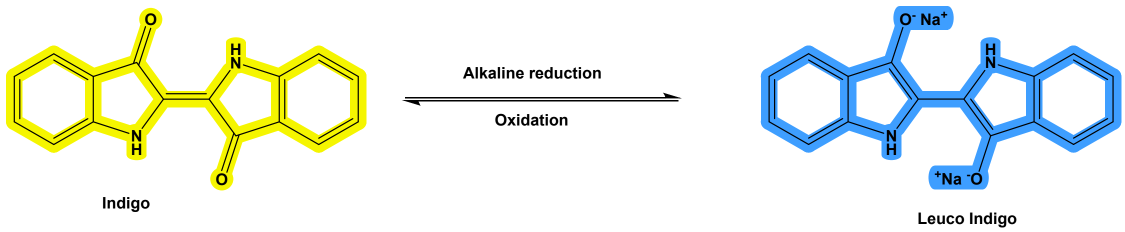

Figure S1. Oxidation-reduction reaction of indigo.

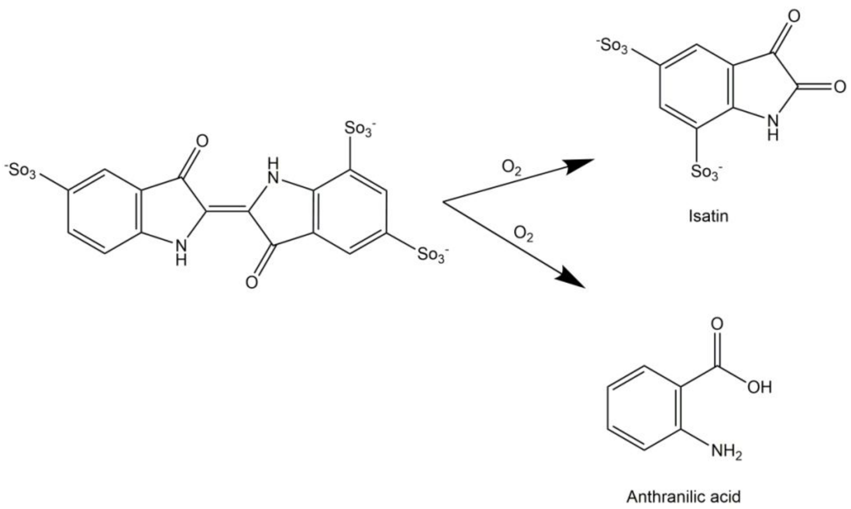

Figure S2. Schematic mechanism of denim bleaching with ozone.

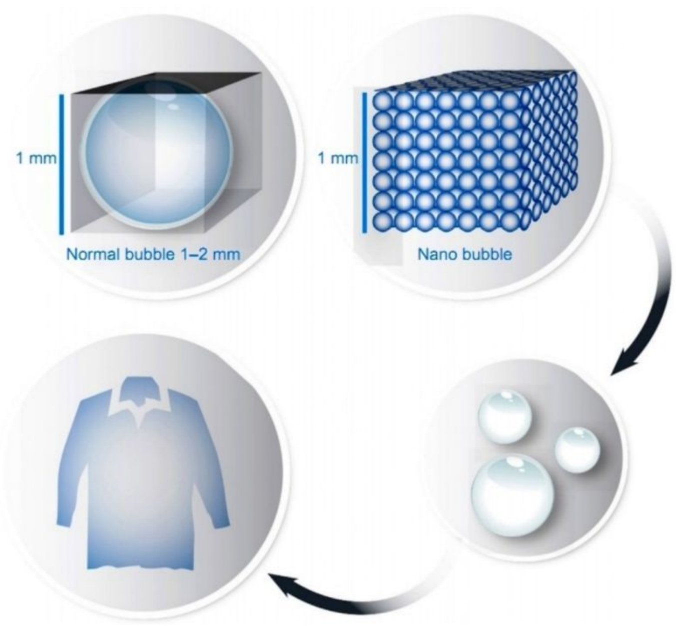

Figure S3. e-flow process for denim.
